# OTEH-12. Assessing Adaptive Responses to Loss of Extrachromosomal DNA Amplification

**DOI:** 10.1093/noajnl/vdab070.051

**Published:** 2021-07-05

**Authors:** Artem Berezovsky, Indrani Datta, Ruicong She, Laura Hasselbach, Laila Poisson, Ana C deCarvalho

**Affiliations:** 1 Henry Ford Health System, Detroit, MI, USA; 2 Wayne State University, Detroit, MI, USA

## Abstract

**Background:**

Oncogene activation through somatic gene amplification happens frequently in GBM, with over 70% of these tumors presenting amplification of at least one putative driver gene, oftentimes in small extrachromosomal circular DNA segments composed of chromatin (ecDNA). A molecularly diverse and representative panel of GBM patient-derived cancer stem-like cells (CSC) and orthotopic mouse xenografts (PDX), which retain the original genomic abnormalities and ecDNA amplifications, was employed to assess adaptive response to the absence of ecDNA amplification.

**Methods:**

We have isolated ecDNA negative cell populations from two patient-derived models. HF3035 harbors a MET amplification and HF3253 harbors a PDGFRA constitutively active genomic rearrangement and extrachromosomal amplification. We conducted paired, whole RNA-sequencing on 20 HF3253 populations (ecDNA+/-: 6 clones from 3 biological replicate PDXs and 4 clones from 4 in vitro technical replicates) and 12 HF3035 population (ecDNA+/-: 6 clones from 3 biological replicate PDXs).

**Results:**

Nonparametric differentially expressed gene (DEG) analysis using NOISeqBio (R/Bioconductor), identified 564 differentially expressed genes (482 upregulated in ecDNA(-)) employing a stringent false discovery rate of 0.05. Genes significantly associated with PDGF stimulation, central carbon metabolism, and H3K27me3 were downregulated in ecDNA(-), while genes significantly associated with astrocytic processes, neuronal differentiation, and EGFR signaling were upregulated in ecDNA(-) (EnrichR). We employed an additive linear model with PDX serving as a blocking factor to compare ecDNA+ and ecDNA- populations in both models (R/edgeR). 2071 genes were upregulated in ecDNA+ PDX specimens and 2365 genes were downregulated. Specifically, E2F targets were highly enriched in ecDNA+ populations, in addition to mRNA pre-processing. ecDNA loss primarily targeted glycogen metabolism, NTRK signaling, and inositol phosphate catabolism.

**Conclusions:**

We have identified PDX-specific and non-specific features to an adaptive response to the loss of ecDNA amplification. Notably, a signature adaptation is an upregulation of seemingly redundant receptor tyrosine kinases.

